# CD8⁺ T Cell Immune Signatures Bridging Systemic Inflammation and Coronary Artery Injury in Kawasaki Disease

**DOI:** 10.1007/s12016-026-09217-8

**Published:** 2026-11-11

**Authors:** Junru Chen, Yidan Zhang, Difan Wang, Ke Li, Zhiyun Liu, Yinghao Wang, Zhenpeng Lu, Guanghui Qian, Panpan Liu, Mingyang Zhang, Jing Li, Haitao Lv, Shuhui Wang

**Affiliations:** 1 Department of Cardiology, Children’s Hospital of Soochow University, Suzhou, 215003 Jiangsu China; 2 Department of Pediatrics, Institute of Pediatric Research, Children’s Hospital of Soochow University, Suzhou, 215003 Jiangsu China; 3 Department of Pediatric Cardiology, Children’s Hospital of Soochow University, No 92, Zhong-nan street, Suzhou, China

**Keywords:** Kawasaki disease, CD8^+^ T cells, Coronary artery injury, Aneurysm, Coronary vasculitis, Immune signatures

## Abstract

Kawasaki disease (KD) is an acute onset, childhood systemic vasculitis with the most important complication being coronary artery injury with aneurysm formation. In the pathogenesis of KD, innate immune overactivation is well recognized, but the role of adaptive immunity in the progression of vascular lesions is not completely understood. A local lesion in the coronary artery is thought to connect to the systemic inflammatory reaction through CD8^+^ T cells. In this review, we discuss evidence from peripheral blood immunophenotyping, single cell transcriptomics, TCR clonal repertoire profiling, human coronary artery pathology and KD like animal models to delineate CD8⁺ T-cell signatures and their proposed pathogenic mechanisms. The circulating CD8^+^ T cells from acute-phase patients have decreased numbers, increased activation markers, skewed subset distribution, and co-existent proliferative and CTL and exhaustion phenotype transcription profiles in conjunction with TCR clonal expansion. CD8^+^ T cells appear to infiltrate the coronary arterial lesions in human autopsy samples and CD8^+^ T cells induce coronary arteritis in murine models. Interestingly, classical perforin-granzyme-mediated cytotoxicity of lesional CD8^+^ T cells is not confirmed in human KD, underscoring a crucial gap between peripheral omics signatures, data in animal models and evidence in human tissues. The in situ effector mechanisms of disease driving CD8+ T cells subsets, and their feasibility as clinical biomarkers, warrant further investigation and may lead to precision risk stratification and targeted immunotherapies in KD related vascular complications, using multicenter prospective cohorts and paired blood and tissue functional assays.

## Introduction

Kawasaki disease (KD) (also called mucocutaneous lymph node syndrome) is an acute systemic vasculitis predominantly affecting children < 5 years, which was first described by Japanese paediatrician Tomisaku Kawasaki in 1967 [1]. Epidemiological surveillance shows that more than 80% of all KD cases are seen in children under 5 years of age with a very high geographical and ethnic variation [2]. Data from multiple countries indicate that the highest incidence of the disease is in infants aged 6–11 months, a period of life when early detection is most critical, although the disease burden is greatest in East Asian populations [2–4]. According to the American Heart Association (AHA) guidelines, the diagnosis of classic KD requires persistent fever for ≥ 5 days together with at least four of the following five clinical features: bilateral nonpurulent conjunctival injection, changes in the lips and oral mucosa, polymorphous rash, changes in the peripheral extremities, and cervical lymphadenopathy [5]. While most children with KD have a favorable post-treatment prognosis, coronary artery lesions are among its most severe complications. Coronary artery aneurysm formation confers long-term risks of thrombosis, coronary stenosis, myocardial ischemia, and sudden death [5–7]. Studies have shown that approximately 25% of untreated children develop coronary artery aneurysms, whereas standard intravenous immunoglobulin (IVIG) therapy can reduce this risk to 4%. However, 10%–20% of affected children are resistant to IVIG, and these patients have a significantly increased risk of coronary artery injury [5].

**Fig. 1:**
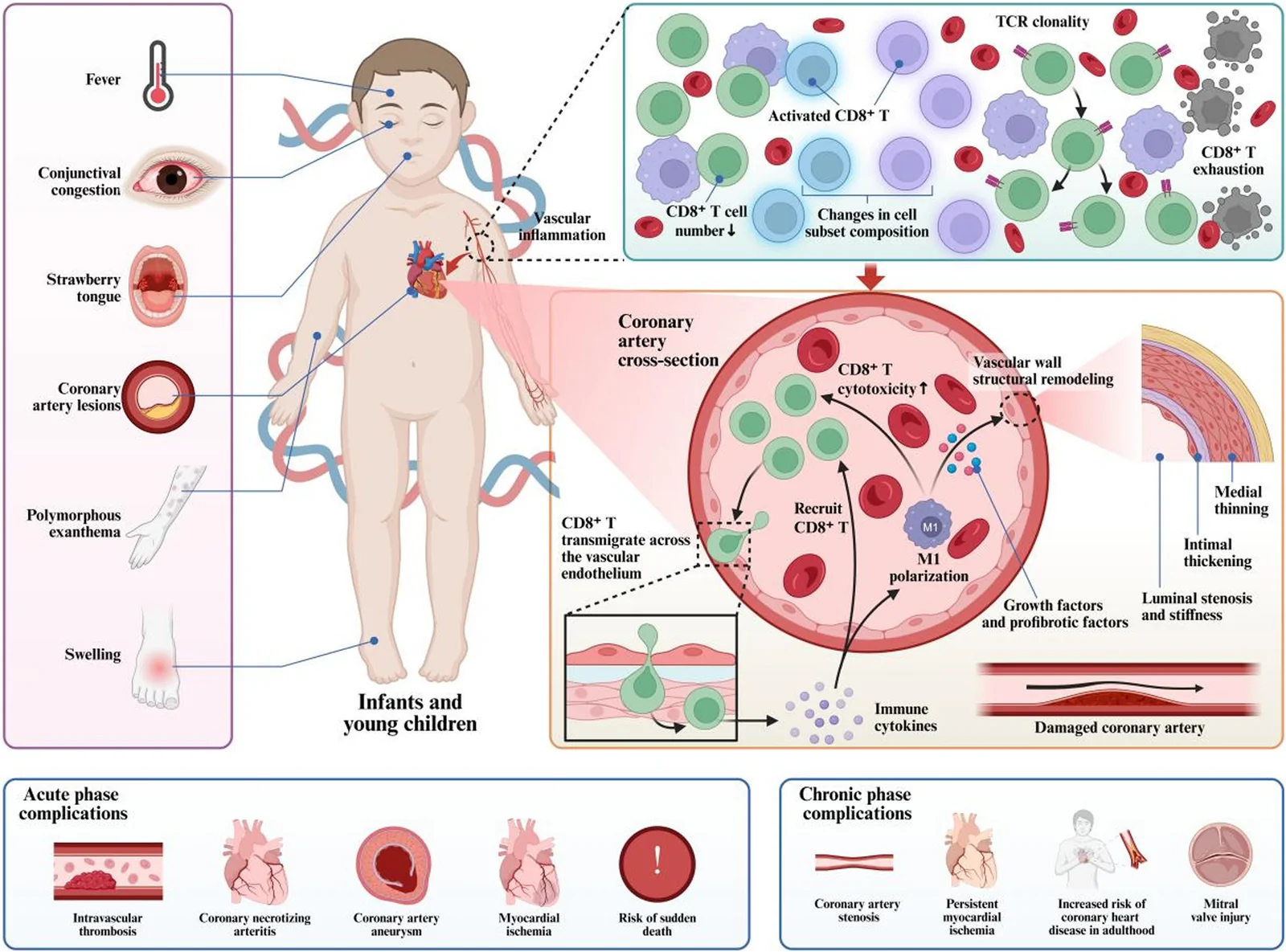
CD8⁺ T cells immune signatures and proposed links between systemic inflammation and coronary artery injury in Kawasaki disease. Kawasaki disease (KD) predominantly affects infants and young children and commonly presents with fever, conjunctival congestion, strawberry tongue, polymorphous exanthema, and extremity swelling, with coronary artery lesions as a key complication. During the acute phase, circulating CD8⁺ T cells may exhibit reduced numbers, enhanced activation, altered subset composition, T-cell receptor (TCR) clonal expansion, and exhaustion-related features. These cells may be recruited to affected coronary arteries and transmigrate across the vascular endothelium. Within the vascular wall, CD8⁺ T cells may display enhanced cytotoxicity and contribute to local vascular inflammation via immune cytokines and crosstalk with macrophages. Persistent inflammation further promotes M1 macrophage polarization, release of growth factors and profibrotic mediators, and subsequent vascular wall structural remodeling, including intimal thickening, medial thinning, luminal stenosis, and increased vascular stiffness. This pathological cascade ultimately leads to acute-phase complications (intravascular thrombosis, coronary necrotizing arteritis, coronary artery aneurysm, myocardial ischemia and risk of sudden death) and chronic-phase complications (coronary artery stenosis, persistent myocardial ischemia, elevated risk of adult-onset coronary heart disease, and mitral valve injury)

The etiology and pathogenic mechanisms of KD are generally considered to involve interactions among genetic, infectious, and immunological factors [8]. The acute phase of KD is characterized by robust innate immune activation, particularly involving the infiltration of neutrophils and macrophages into vascular tissues [9]. Concurrently, adaptive immune abnormalities are evident, with immunoglobulin A (IgA)-producing plasma cell infiltration in the vascular wall serving as a distinctive histopathological feature associated with disease severity [10]. However, with the development of technology, it has been possible to obtain a more detailed picture of the immune microenvironment of KD. CD8⁺ T cells are important effector cells of adaptive immunity that can not only differentiate into CTLs to mediate destruction of infected cells by releasing granzymes and perforin, but also further regulate immune responses by secreting interferon-gamma (IFN-γ) and tumour necrosis factor-alpha (TNF-α) [11]. CD8^+^ T cells are now thought to be involved in a number of diseases such as infections, auto-immune diseases and vasculitis. KD follows a characteristic clinical course. The acute febrile phase typically lasts 1 ~ 2 weeks and is followed by a subacute phase (approximately weeks 2 ~ 4), during which fever usually subsides but the risk of coronary artery aneurysm formation and progression remains significant; the disease subsequently enters a convalescent phase that may last weeks to months [12]. Coronary artery lesions may develop and evolve during the acute and subacute phases, and pathological evidence indicates that vascular injury can progress even after the resolution of fever [13]. Although these findings have been made, there is still not a clear picture of how the activity of CD8^+^ T cells links to the development of coronary artery aneurysms, and additional studies are needed to understand the precise mechanisms involved and to determine the potential for targeted treatments [14, 15]. This review focuses on the immune characteristics of CD8⁺ T cells and systematically summarizes the research progress of their involvement in the pathogenesis of KD in the acute phase, aiming to explore how these cells link inflammation with vascular injury, so as to provide a new perspective for mechanism research and clinical intervention. The potential progression from peripheral CD8⁺ T cells alterations to recruitment into affected coronary arteries, local vascular inflammation, subsequent structural remodeling, and acute and chronic phase complications is summarized in Fig. 1.

## Immunological Basis and Inflammatory Microenvironment of KD

### Immunological Basis of K*D*

The etiopathogenesis of KD is not fully understood and is a challenge in pediatric cardiology [8]. It is now believed that KD is the result of an abnormal immune reaction to infectious or environmental events in a genetically predisposed host [16, 17]. Epidemiological evidence including the spread of the infection from person to person with an unknown agent and genetic predispositions of the host that control the intensity of the inflammation [18] support this multifactorial model. The genetic susceptibility is a key factor in the onset of disease and clinical appearance, especially with regard to coronary artery lesions (CALs) [19]. Several polymorphisms have been identified in the inositol 1, 4, 5-trisphosphate 3-kinase C (ITPKC) gene that have been consistently linked to increased KD susceptibility and a higher risk of CAL formation in various populations [20, 21]. Furthermore, variants in caspase-3 (CASP3) contribute to apoptosis dysregulation, which may prolong inflammation and exacerbate vascular damage [22]. Given that the incidence of coronary artery lesions in untreated KD patients is about 25%, and the rate of IVIG resistance is as high as 10%-20%, it is still a key direction for future research to clarify the causal chain between infection, CD8^+^ T cell dysregulation and vascular injury [23, 24].

In addition to innate immunity, adaptive immune mechanisms such as B-cell signaling and T-cell co-stimulation play a crucial role in KD pathogenesis [25]. B lymphoid tyrosine kinase (BLK) and cluster of differentiation 40 (CD40) are genome-wide association studies (GWAS) susceptibility genes, and the risk alleles are associated with changes in function of B cells and increased immune activation in the acute phase [26, 27]. Additionally, the FCGR2A H131R polymorphism influences Fc receptor-mediated responses, affecting both intravenous immunoglobulin (IVIG) treatment efficacy and coronary outcomes [28]. Yet, heterogeneity of genetic associations between ethnicities and incomplete knowledge of the interaction between genes and the environment remain controversial. Collectively, these findings suggest that genetic susceptibility may shape the magnitude and characteristics of immune responses to environmental or infectious triggers, providing an important background for the inflammatory microenvironment observed in KD.

### Formation and Characteristics of the Inflammatory Microenvironment in KD

KD is characterized by a sustained, dysregulated inflammatory response during the acute phase that fundamentally differs from self-limiting physiological inflammation [29]. This pathological state involves complex interactions between innate and adaptive immune cells, leading to a failure in resolution mechanisms that typically terminate acute inflammation [10, 30–34].

Endothelial activation and subsequent vascular microenvironment remodeling are central to the coronary artery injury observed in KD [19]. Recent evidence indicates that hypoxia-inducible factor 2 (HIF2) activation in the cardiac vasculature leads to arterial dilation, thrombosis and inflammation, which mirrors important aspects of pathological severe KD [6]. One of the characteristics of this inflammatory environment is the constant presence of pro-inflammatory cytokines such as interleukine-1 beta (IL-1β), interleukine 6 (IL-6) and tumour necrosis factor alpha (TNF-α) in the peripheral blood of patients in the acute phase [35]. These systemic markers not only reflect disease activity but also correlate with the severity of coronary artery lesions, indicating their role in propagating vascular damage.

Chemokines such as C-X-C motif chemokine ligand 10 (CXCL10) and C-C motif chemokine ligand 2 (CCL2) are significantly upregulated in acute KD and facilitate the recruitment of immune cells to the coronary arteries [36]. Importantly, the CXCL10/CXCR3 axis has been suggested to favor Th1 cell infiltration and to maintain cardiac inflammation and is therefore a potential target for modulator therapy [36]. Coronary artery lesions are histopathologically characterized by ongoing inflammatory cell infiltration and local over-expression of the same mediators, namely IL-1β, IL-6, TNF-α, CXCL10 and CCL2 [37]. This local amplification of the inflammatory signals forms a positive feedback loop which helps to maintain vascular wall inflammation and is thought to contribute to tissue remodeling and aneurysm formation [5, 10, 15, 38, 39].

In addition, sustained inflammatory stimulation leads to the activation of vascular endothelial cells, which subsequently increases the expression of adhesion molecules such as intercellular adhesion molecule 1 (ICAM-1) and vascular cell adhesion molecule 1 (VCAM-1), thereby facilitating inflammatory cell adhesion and infiltration [40]. This endothelial dysfunction facilitates the recruitment of monocytes and macrophages into the vascular wall, where they release matrix metalloproteinases that degrade the extracellular matrix and disrupt the internal elastic lamina [10, 41, 42]. Such histopathological changes initiate a cascade of vascular remodeling that can progress from necrotizing arteritis to chronic luminal stenosis or dilation [30]. At the same time, the immune microenvironment of acute KD is highly disregulated with respect to adaptive immunity, in particular with respect to CD8^+^ T cells [43]. In the absence of timely intervention, this ongoing inflammatory state causes formation of coronary artery aneurysms in about a quarter of patients that do not receive treatment [5]. The risk of this complication is greatly lowered by early treatment with intravenous immunoglobulin, but the exact association between long-term vascular outcomes and specific subsets of immune cells remains unclear, and more studies are needed on targeted immunomodulatory therapies.

Thus, the immune microenvironment created during the acute phase of KD induces recruitment of immune cells to various locations, such as the coronary arteries, by the release of pro-inflammatory mediators, expression of chemokines and activation of vascular endothelial cells. This persistent inflammatory state also provides an immunological basis for the abnormal activation, functional alterations, and tissue infiltration of CD8⁺ T cells [23, 41, 44].

## Immunological Characteristics of CD8⁺ T Lymphocytes in KD

CD8⁺ T cells are important effector cells of adaptive immunity, and their functional status requires comprehensive evaluation across multiple immunological dimensions. The relevant findings are summarized in Table 1. Current evidence suggests that CD8⁺ T cells in patients with KD exhibit complex heterogeneity and dynamic changes.

**Table 1:** Major immunological characteristics of CD8⁺ T cells in Kawasaki disease

Immunological feature	Marker/Method	Key findings	Interpretation/Main limitations	References
Peripheral blood cell numbers	CD8⁺ T cell​ proportion and absolute count	Reduced during the acute phase and increased after treatment and during convalescence	Mechanisms remain unclear; limited specificity for KD.	Wang et al. [45]; Choi et al. [46]; Xie et al. [47]; Lee et al. [48]
Early activation	CD69	Increased proportion of CD69⁺CD8⁺ T cells during the acute phase.	Indicates early activation; clinical significance requires validation.	Ehara et al. [50]
HLA-DR-related activation	CD8⁺HLA-DR⁺ T cell​ proportion; CD8⁺HLA-DR⁺/CD8⁺CD69⁺ ratio	Elevated in acute KD; higher in IVIG nonresponders.	Suggests persistent or later-stage activation; possibly associated with IVIG nonresponse; independent predictive value remains unestablished.	Ye et al. [23]
CD25-related activation and regulation	CD25 and CD69; in vitro activin A treatment	CD25 and CD69 expression increased during the acute phase and decreased after activin A treatment.	May reflect activin A-mediated regulation; limited by the small sample and in vitro design.	Wu et al. [44]
Differentiation subset numbers	CD45RA/CD27 flow cytometry	Subset proportions were unchanged, whereas absolute counts of multiple subsets decreased during the acute phase.	Acute-phase changes mainly involve reduced subset counts.	Ehara et al. [50]
Differentiation subset composition	CCR7, SELL, and S100A4 transcriptional markers	Increased naïve and decreased effector-memory cell proportions during the acute phase.	Indicates altered CD8⁺ T cell​ subset composition.	Wang et al. [45]
Proliferative CD8⁺ T cell subset	MKI67-high CD8_Pro subset	Stronger cytotoxicity-related features in patients with coronary artery lesions.	Coronary artery infiltration and direct pathogenic involvement remain unclear.	Chen et al. [64]
Exhaustion-like features	Exhaustion-related genes and cytotoxicity scores	Exhaustion-related features coexisted with high cytotoxicity scores in some effector CD8⁺ T cells.	The two programs may coexist transcriptionally; functional validation is lacking.	Feng et al. [65]
Restricted effector differentiation	Single-cell pseudotime analysis	Possible restriction of effector differentiation in IVIG-nonresponsive patients with coronary artery lesions.	Based mainly on single-cell analysis; independent validation is needed.	Zheng et al. [68]
TCR clonal features	TCR sequencing and TCR Vβ repertoire analysis	Clonal TCRs were enriched in the GZMB-high effector-memory subset. Clonality may increase after IVIG.	Indicates TCR clonal expansion.	Choi et al. [101]; Wang et al. [45]; Mancia et al. [69]

*CCR7* C-C motif chemokine receptor 7, *CD* cluster of differentiation, *CD8_Pro* proliferating CD8⁺ T-cell subset, *GZMB* granzyme B, *HLA-DR* human leukocyte antigen-DR, *IVIG* intravenous immunoglobulin, *KD* Kawasaki disease, *MKI67* marker of proliferation Ki-67, *S100A4* S100 calcium-binding protein A4, *SELL* selectin L, *TCR* T-cell receptor, *Vβ* variable region of the TCR β chain

### Changes in CD8⁺ T-Cell Numbers

The acute phase of KD is frequently accompanied by marked alterations in the composition of peripheral blood immune cells. Multiple studies have reported an overall reduction in T lymphocytes, with a trend toward a lower proportion of circulating CD8⁺ T cells in affected children than in healthy children [45]. However, compared with children with other febrile illnesses, the magnitude of the reduction in CD8⁺ T cells often does not differ significantly [46], implying that such alterations are shaped jointly by KD-specific immune responses and nonspecific acute systemic inflammation. The peripheral CD8⁺ T-cell reduction is hypothesized to stem from cellular redistribution toward inflamed tissues; nevertheless, coronary pathological observations and immune-cell atlas data only offer indirect support for this notion and cannot confirm that circulating CD8⁺ T cells directly migrate into coronary arterial lesions [41, 47].

CD8⁺ T-cell numbers also change dynamically with disease progression and therapeutic intervention. An early study found that the proportion of CD8⁺ T cells was reduced before treatment in children with KD but showed a significant rebound approximately 4 days after treatment with IVIG combined with aspirin [48]. In a longitudinal study of 68 children with KD, Choi et al. also observed significant fluctuations in the proportion of peripheral blood CD8⁺ T cells before IVIG administration, after treatment, and during convalescence [46]. Collectively, these data indicate that acute-phase peripheral CD8^+^ T cell reduction is transient and reversible upon inflammation resolution, yet its precise mechanisms, including cell redistribution, proliferation, apoptosis and subset remodeling, remain unclear.

### Activation Characteristics of CD8⁺ T Cells

Dysregulation of T lymphocyte subsets, particularly the expansion and abnormal activation of CD8^+^ T cells, has emerged as a hallmark immunopathological feature during the acute phase of KD [15]. Histopathological analyses of vasculitis lesions reveal significant infiltration of CD8^+^ T cells expressing cytotoxic proteins such as granzyme B and TIA-1, suggesting that these cells contribute directly to endothelial injury and the subsequent development of coronary artery lesions [14, 49]. It is important to emphasize that the expression of cytotoxic effector molecules and the corresponding transcriptional signatures reflect the cytotoxic-related state of circulating CD8^+^ T cells and do not by themselves demonstrate effective degranulation or direct killing of vascular cells; direct cytotoxic effector mechanisms have not been demonstrated in human coronary artery lesions. Current studies have reported an increased proportion of CD8⁺human leukocyte antigen-DR (HLA-DR)⁺ T cells, accompanied by altered expression of activation markers such as CD69 and CD25, in patients with acute KD [23, 44, 50]. Ehara et al. found that the proportion of CD69⁺CD8⁺ T cells was markedly increased in children with acute KD compared with healthy children and children with other febrile illnesses, suggesting prominent early activation of peripheral CD8⁺ T cells [50]. Surface markers such as CD69 and HLA-DR are widely recognized in immunology as indicators of early and late-stage T-cell activation, respectively, although their specific kinetics in KD warrant further study [50].

The relationship between HLA-DR-related phenotypes and the response to IVIG treatment has also received considerable attention. Studies have shown that the proportion of CD8⁺HLA-DR⁺ T cells was increased in children with acute KD compared with healthy controls. Furthermore, the ratio of CD8⁺HLA-DR⁺ to CD8⁺CD69⁺ T cells is significantly higher in IVIG-nonresponsive patients than in IVIG-responsive counterparts, indicating that an imbalance between sustained activation and early activation markers may predict treatment failure [23]. These findings suggest that enhanced persistent or later-stage activation of CD8⁺ T cells may be associated with a poor response to IVIG and may reflect insufficient control of inflammatory activity [23]. Although current clinical guidelines do not yet incorporate these specific T-cell metrics for risk stratification, ongoing research into advanced biomarkers, including immune phenotyping, highlights the need for further multicenter validation to establish their clinical role [51].

In addition, a small-sample study observed increased expression of CD69 and CD25 on CD8⁺ T cells in children during the acute phase. The expression of both markers decreased following the addition of activin A in vitro, suggesting that activin A may be involved in regulating CD8⁺ T-cell activation [44]. Several biomarkers have been linked to IVIG resistance; the value of activation markers, such as CD69 and HLA-DR, of CD8^+^ T cells in predicting IVIG response is a subject of current research. In the future, longitudinal immunophenotyping should be undertaken to see if these activation markers can be used as a reliable marker for treatment response and long-term cardiovascular risks in children [52].

### Differentiation Subsets and Compositional Changes in CD8⁺ T Cells

Based on their differentiation and functional status, CD8^+^ T cells can be divided into various subsets: naïve, effector and memory cells. This classification reflects the dynamic process, during which CD8^+^ T cells are activated, clonally expanded and differentiate into functional effector cells, and long-term immunological memory is established, after stimulation with antigens [53, 54]. Besides alteration in the absolute number of peripheral blood CD8^+^ T cells during the acute phase of KD, the intra-subset distribution of the cells could also be changed [45].

The classification of CD8⁺ T-cell subsets exhibits significant heterogeneity across immunological studies, reflecting the complex and dynamic nature of adaptive immunity [55]. While traditional subset-based systems have long been used to categorize these cells, recent advances in single-cell technologies reveal an astounding degree of phenotypic and transcriptional diversity that challenges rigid nomenclature [56]. The differentiation states of naïve and memory subsets can be tracked during infection using a phenotypic approach that is widely used, which is based on co-expression of CD45RA and CD27 [57]. Other strategies, however, use transcriptional markers like CCR7, SELL (L-selectin), and S100A4 to identify central-memory identities with associated homing abilities and functional capacities [45, 50]. This methodological discordance means that subsets bearing similar names across different studies are not necessarily defined by identical criteria, thereby impeding direct cross-study comparability and meta-analytic integration [58]. Such inconsistencies in immunophenotyping panels and gating strategies can lead to divergent interpretations of immune status, particularly when assessing age-related shifts where naïve CD8⁺ T-cell proportions decline while effector-memory subsets expand [59].

Under these different classification systems, current studies have not reported entirely consistent changes in CD8⁺ T cell​ subsets during the acute phase of KD. High-resolution profiling of CD8^+^ T cell​ subsets in the acute phase using single-cell RNA sequencing (scRNA-seq) and flow cytometry has shown a relative increase in naïve and a relative decrease in effector-memory cells, compared with healthy controls [45]. Recent transcriptomic data indicate that it is the relative change of CD8^+^ T cell​ subsets in the acute phase that is important, making the choice of detection platform crucial for the interpretation of immune phenotypes. Different CD8^+^ T cell​ dynamics have been reported and can perhaps be explained by differences in study populations and analytical techniques [60]. Notably, excessive CD8⁺ T cell​ activation has been associated with IVIG resistance, indicating that persistent immune dysregulation during convalescence may reflect incomplete restoration of naïve and effector-memory homeostasis in non-responders [23].

Overall, current findings suggest that the reduction in peripheral blood CD8⁺ T cells during the acute phase may be accompanied by subset-specific changes in cell numbers or composition; however, no consistent pattern of these changes has yet been established.

### Effector Programs and Clonal Features of CD8⁺ T Cells

Effector CD8⁺ T cells can produce cytokines such as IFN-γ and TNF-α and express effector molecules including perforin and granzymes, providing the functional basis for their participation in immune responses and target-cell killing [61]. Perforin 1 (PRF1) and granzyme B (GZMB) are core effectors of cytotoxic granule‑mediated target-cell killing. PRF1 generates target-cell plasma-membrane pores, inducing membrane repair and endocytosis to facilitate GZMB cytosolic entry and subsequent apoptotic activation [62]. In addition to effector molecules that directly mediate target-cell killing, cytotoxic programs also involve molecules associated with granule release. Among these, natural killer cell granule protein 7 (NKG7) participates in the degranulation of cytotoxic lymphocytes [63].

Single-cell RNA sequencing studies have characterized the cytotoxicity-related transcriptional state of CD8⁺ T cells in KD by analyzing the expression profiles of key effector molecules, including PRF1, GZMB, GZMK, NKG7, and GNLY [64, 65]. These analyses highlight that there is a high level of heterogeneity in these effector states, with a high MKI67 expressing subset (CD8_Pro) possessing greater cytotoxic features, especially in CAL [66] children. It is controversial, however, what the functional implications of these signatures are because high gene expression does not always equate to effective degranulation or target-cell killing capacity [67]. In addition, pseudotime analysis suggests that patients with CAL refractory to IVIG treatment have defects in their CD8^+^ effector T cell differentiation, and evidence shows that cytotoxic programs can coexist with exhaustion-like transcriptional programs in refractory cases [68].

Thus, the current data should be viewed as a transcriptionally active state associated with cytotoxicity, and targeting the pathway needs to be better understood. Future studies should focus on functional assays to evaluate degranulation and killing activity of these transcriptionally active CD8 T cells and establish whether they are directly involved in the vascular injury and add to IVIG resistance [66].

TCR repertoire analysis has further revealed clonal selection patterns in CD8⁺ T cells. Studies have shown that memory CD8⁺ T cells expressed relatively high levels of cytotoxicity-associated genes such as GZMK and GZMB, with cells carrying clonally expanded TCRs predominantly enriched in the GZMB-high effector-memory subset [45]. In addition, TCR clonality increased after IVIG treatment in most patients compared with pretreatment levels, without an apparent bias in T-cell receptor alpha variable (TRAV) or T-cell receptor beta variable (TRBV) gene usage. Together with previous analyses of the TCR Vβ repertoire, these findings suggest that the T-cell response in KD may be more consistent with the characteristics of a conventional antigen-driven immune response [45, 69]. Although TCR clonal expansion in KD suggests antigen-driven selection, the nature of the antigens recognized by the expanded CD8⁺ T-cell clones remains unknown. Current data do not allow us to determine whether these clones recognize specific pathogen-derived epitopes, self-antigens, or expand through bystander mechanisms. Clarifying the antigen specificity of the clonally expanded CD8⁺ T cells is therefore an important priority for future research.

## Potential Mechanisms by Which CD8⁺ T Cells Contribute to Vascular Inflammation and Coronary Artery Injury

During the acute phase of KD, inflammatory mediators can activate the vascular endothelium and enhance the expression of adhesion molecules, thereby providing conditions that facilitate the recruitment of immune cells to the affected coronary artery wall [70]. Human pathological studies and animal models have provided spatial and functional evidence, respectively, for the entry of CD8⁺ T cells into affected coronary arteries and their involvement in the development of vasculitis [41, 71]. These findings suggest that CD8⁺ T cells may contribute to coronary artery inflammation and injury through recruitment to the coronary arteries, local effector functions, and interactions with other immune cells. However, the specific mechanisms involved and their roles in human disease require further validation. The relevant potential mechanisms and supporting evidence are summarized in Table 2.

**Table 2:** Potential mechanisms and evidence for CD8⁺ T-cell involvement in coronary artery injury in Kawasaki disease

Potential mechanism/function	Key factors	Main evidence	Interpretation/Main limitations	References
Coronary artery infiltration	Transmural CD8⁺ T-cell infiltration with macrophage accumulation	CD8⁺ T cells outnumbered CD4⁺ T cells in acute coronary artery aneurysm tissues.	Direct histological evidence; limited to fatal cases.	Brown et al. [41]
Inflamed endothelium-mediated adhesion and migration	IL-1β, ICAM-1, VCAM-1	IL-1β and acute-phase serum enhanced endothelial adhesion molecule expression.	May facilitate CD8⁺ T-cell recruitment; direct evidence is lacking.	Armaroli et al. [70]; Inoue et al. [74]; Nourshargh and Alon [75]; Ley et al. [76]
Chemokine-directed recruitment	CXCL9, CXCL10, CXCR3	CXCL9 and CXCL10 were elevated in acute KD; CXCR3 can mediate CD8⁺ T-cell chemotaxis	Provides a possible chemotactic background; not directly demonstrated in KD coronary arteries.	Griffith et al. [111]; Hosaka et al. [112]; Groom and Luster [77]; Kobayashi et al. [14]
Local antigen presentation and maintenance of T-cell responses	Dendritic cells, macrophages, interferon signaling	Dendritic cells colocalized with CD3⁺ T cells in coronary arteries; related signals persisted in chronic lesions.	May sustain local T-cell responses; evidence is not CD8⁺ T-cell-specific.	Yilmaz et al. [79]; Rowley et al. [80]
Potential cytotoxic effects	Perforin–granzyme Fas–FasL pathways	Peripheral blood CD8⁺ T cells showed cytotoxicity-related transcriptional features.	Classical direct cytotoxicity remains unproven in human coronary arteries.	Chen et al. [64]; Feng et al. [65]; Guzman-Cottrill et al. [82]; Inoue et al. [74]
Noncytolytic inflammatory effects	IFN-γ, TNF-α	Affect endothelial permeability, migration, and barrier function.	Indirect support; production by coronary-infiltrating CD8⁺ T cells is unproven.	Hamann et al. [61]; Ferro et al. [83]; Ng et al. [84]; Sakata et al. [90]
Persistent inflammation and remodeling	T-cell-related inflammatory signals	Persist in chronic coronary lesions.	May sustain local inflammation; CD8⁺ T-cell-specific evidence is lacking.	Rowley et al. [80]

*CD* cluster of differentiation, *CXCL9* C-X-C motif chemokine ligand 9, *CXCL10* C-X-C motif chemokine ligand 10, *CXCR3* C-X-C motif chemokine receptor 3, *Fas* Fas cell surface death receptor, *FasL* Fas ligand, *ICAM-1* intercellular adhesion molecule 1, *IFN-γ* interferon-gamma, *IL-1β* interleukin-1 beta, *KD* Kawasaki disease, *TNF-α* tumor necrosis factor-alpha, *VCAM-1* vascular cell adhesion molecule 1

### Endothelium-Driven Recruitment and Intra-Lesional Reactivation of CD8⁺ T Cells

In order to infiltrate the coronary artery wall, circulating CD8^+^ T cells need to undergo a series of events such as chemotaxis, endothelial adhesion and transendothelial migration. CD8^+^ T cell infiltration in the vascular wall is a characteristic feature of immune-mediated vasculitis in KD, and is sometimes associated with the severity of the tissue damage [72]. In coronary artery aneurysm tissues from eight children who died during the acute phase, CD45RO⁺ memory T cells were observed to infiltrate across the vascular wall, with CD8⁺ T cells predominating over CD4⁺ T cells and accompanied by macrophage accumulation [14, 72]. These co-localization studies indicate that both cytotoxic T lymphocytes and myeloid cells could play a role in local inflammation and could act synergistically to cause vascular wall damage. In some cases, patchy infiltrates were also seen in the myocardium, which are mainly composed of CD8^+^ T cells and could be a secondary inflammatory spillover or the direct involvement of the myocardium [72]. However, these findings are only from acute-phase fatal cases, however, and these cases constitute less than 1% of all the Kawasaki disease admissions, but offer unique insights into end-stage immune-mediated vascular injury [73]. While tissue infiltration confirms the presence of CD8⁺ T cells in affected sites, it alone cannot establish their specific pathogenic role or causal relationship with aneurysm formation.

The inflammatory environment during the acute phase of KD may establish the conditions required for CD8⁺ T-cell recruitment by activating the vascular endothelium. Monocyte-derived IL-1β can induce inflammatory activation of human coronary artery endothelial cells and promote the expression of adhesion molecules such as ICAM-1 and VCAM-1 [70]. In addition, serum from children with acute KD has been shown to enhance ICAM-1 expression in human umbilical vein endothelial cells (HUVECs), further indicating that the circulating inflammatory environment during the acute phase can enhance the endothelial adhesive phenotype [74]. General studies of lymphocyte migration have shown that interactions between lymphocyte function-associated antigen 1 (LFA-1) and ICAM-1, as well as between very late antigen 4 (VLA-4) and VCAM-1, can contribute to T-cell adhesion to the endothelium and transendothelial migration [75, 76]. However, these processes have not been directly validated in coronary artery-infiltrating CD8⁺ T cells in KD.

Chemokines may further influence the direction of CD8⁺ T-cell migration [40]. Levels of C-X-C motif chemokine ligand 9 (CXCL9) and CXCL10 are elevated during the acute phase of KD and decline as inflammation subsides after treatment [41]. General studies have shown that subsets of activated or memory CD8⁺ T cells can express C-X-C motif chemokine receptor 3 (CXCR3) and migrate toward CXCL9 and CXCL10, thereby trafficking to inflamed tissues [77, 78]. Accordingly, fluctuating CXCL9 and CXCL10 levels in acute KD may establish a chemotactic milieu for CD8⁺ T-cell redistribution between circulation and inflamed tissues.

**Fig. 2:**
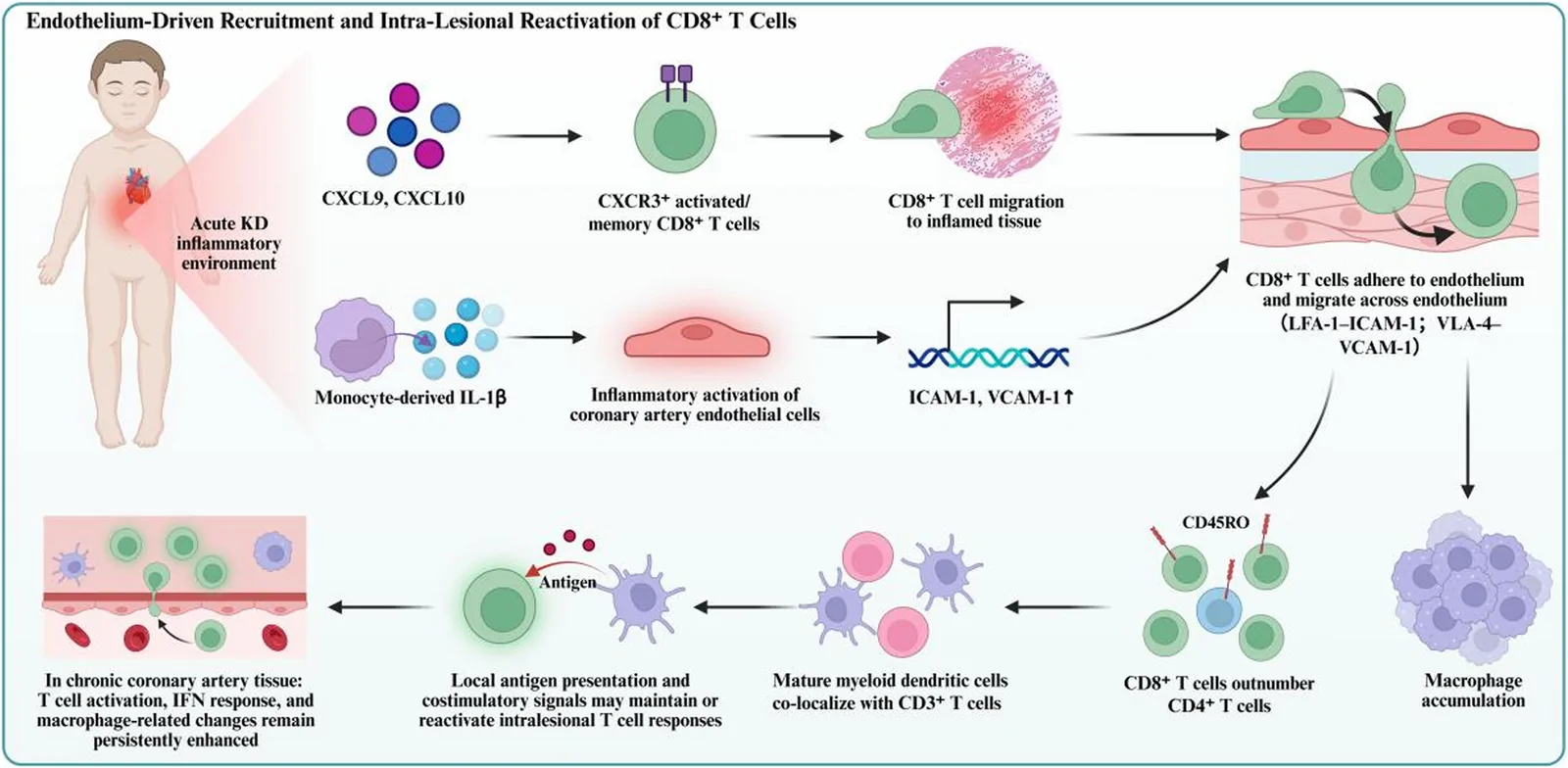
Endothelium-driven recruitment and intra-lesional reactivation of CD8⁺ T cells in Kawasaki disease. Schematic diagram illustrating hypothetical steps for CD8⁺ T-cell recruitment and reactivation within coronary arterial lesions during Kawasaki disease (KD). In the acute KD inflammatory milieu, monocyte-derived IL-1β induces endothelial activation and up-regulation of adhesion molecules ICAM-1 and VCAM-1. Meanwhile, elevated CXCL9 and CXCL10 serve as chemokines to recruit CXCR3-expressing activated/memory CD8⁺ T cells. Circulating CD8⁺ T cells adhere to endothelium via LFA-1-ICAM-1 and VLA-4-VCAM-1 interactions and subsequently complete transendothelial migration. Within vascular lesions, CD8⁺ T cells outnumber CD4⁺ T cells, accompanied by macrophage accumulation. Mature myeloid dendritic cells colocalize with T-cell subsets and provide local antigen presentation and costimulatory signals to sustain or reactivate intra-lesional CD8⁺ T-cell responses. In chronic coronary artery lesions from KD patients, T-cell activation signatures, interferon responses and macrophage-related alterations remain persistently enhanced

Upon infiltrating the vascular wall, CD8⁺ T-cell activation may be sustained by local antigen presentation and costimulatory signals. Mature myeloid dendritic cells accumulate and colocalize with CD3⁺ T cells in KD coronary lesions, pointing to local antigen-presenting cells mediating T-cell-response activation and maintenance [79]. In chronic KD coronary artery tissues, signals associated with T-cell activation and interferon responses remain enhanced and are accompanied by macrophage-related changes, suggesting that persistent interactions may occur between myeloid cells and T cells [80]. It should be noted that, although CD8^+^ T cells are present within coronary artery lesions, direct evidence that circulating CD8^+^ T cells migrate to the coronary artery wall is currently lacking; the recruitment of CD8^+^ T cells to the coronary arteries therefore remains a hypothesis supported by indirect evidence. Therefore, local antigen presentation and interactions with myeloid cells can currently only be regarded as potential mechanisms that maintain coronary artery CD8⁺ T-cell responses and require further validation. Collectively, these multistep processes of chemotaxis, endothelial adhesion, transendothelial migration and local re-activation are schematically illustrated in Fig. 2.

### Putative Direct Cytotoxic Damage Mediated by CD8^+^ T Lymphocytes

Single-cell studies of KD have shown that some CD8⁺ T cells exhibit a cytotoxicity-related transcriptional state, suggesting that they possess the molecular basis required to exert direct effector functions [45, 64, 65]. Activated cytotoxic CD8⁺ T cells can generally release cytotoxic granules through the perforin–granzyme pathway or induce target-cell apoptosis through the Fas–Fas ligand (FasL) death receptor pathway [62, 81]. In a KD-like mouse model induced by Lactobacillus casei cell wall extract (LCWE), CD8⁺ T-cell infiltration was observed within coronary artery lesions. Depletion of CD8⁺ T cells with an anti-CD8 antibody markedly reduced pericoronary and myocardial inflammation, whereas adoptive transfer of CD8⁺ T cells into mice lacking mature lymphocytes restored coronary arteritis and myocarditis [71]. These intervention experiments provide robust evidence that CD8⁺ T cells promote the development of vasculitis in this murine context​.

Despite these findings in animal models, existing human pathological evidence has not clearly demonstrated direct cytotoxic processes mediated by CD8⁺ T cells in coronary artery lesions. One study failed to detect clear expression of perforin and GZMB in CD8⁺ T cells infiltrating coronary artery lesions, indicating that the perforin-granzyme pathway may not be the primary driver of vascular injury in patients [82]. Furthermore, serum from patients with acute KD did not induce Fas expression in cultured HUVECs, and direct interactions between CD8⁺FasL⁺ cells and Fas⁺ vascular wall cells have not yet been demonstrated in human tissues [74].

**Fig. 3:**
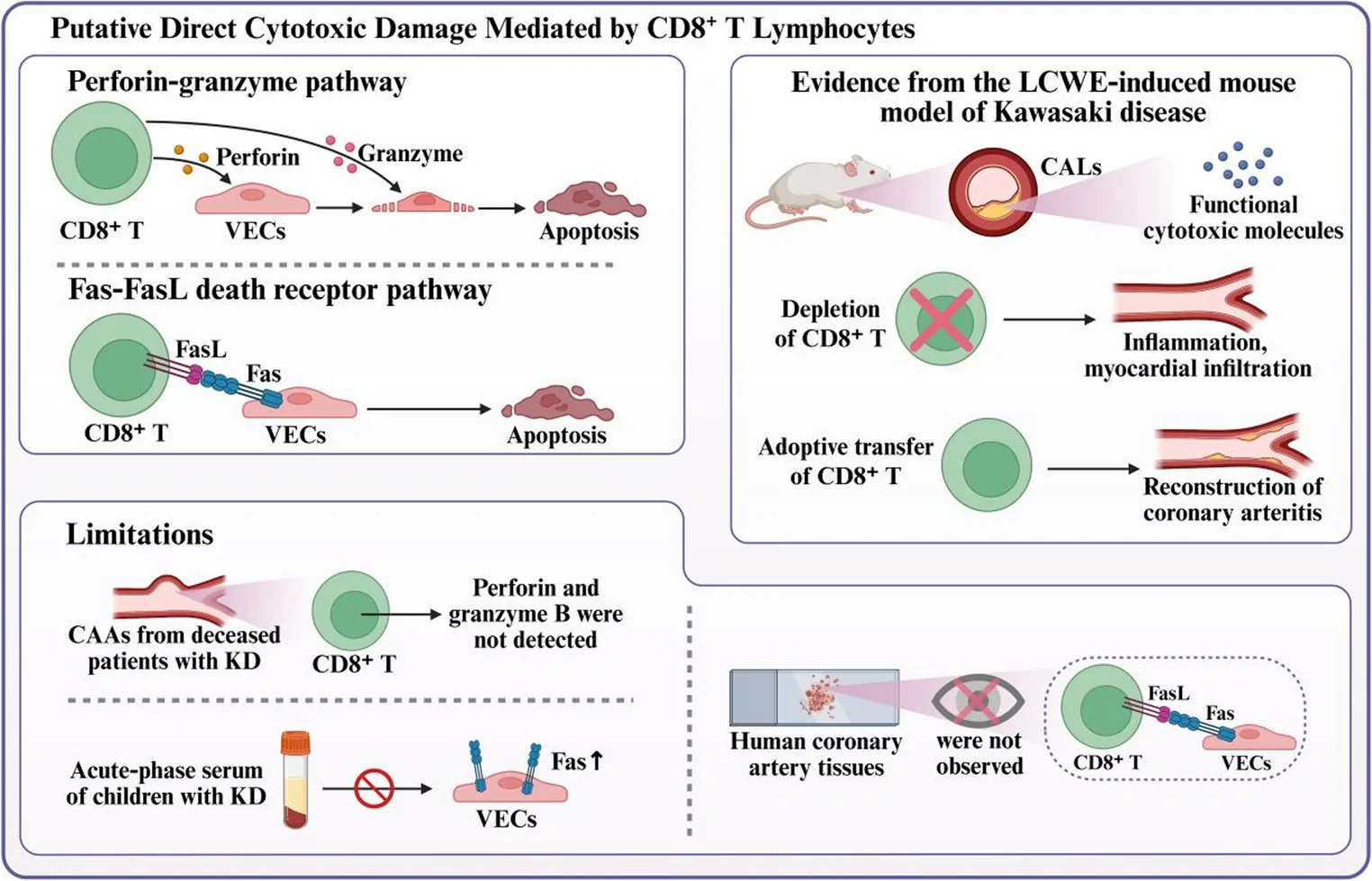
Putative direct cytotoxic damage mediated by CD8⁺ T lymphocytes in Kawasaki disease-associated vasculitis. This schematic summarizes CD8⁺ T-cell-mediated cytotoxic pathways and the discrepancy between animal and human findings in KD. Activated cytotoxic CD8⁺ T cells can theoretically injure vascular endothelial cells (VECs) through the perforin-granzyme pathway to trigger target-cell apoptosis, or via the Fas-FasL death-receptor signaling. Studies using the Lactobacillus casei cell-wall extract (LCWE) mouse model of KD support the pathogenic role of CD8⁺ T cells: CD8⁺ T-cell depletion ameliorates coronary and myocardial inflammation, whereas adoptive transfer of CD8⁺ T cells ​restores vasculitis. Nevertheless, human pathological evidence does not confirm such direct cytotoxicity. Perforin and granzyme B are barely detectable in lesion-infiltrating CD8⁺ T cells from human coronary artery aneurysm tissues. In addition, acute-phase KD serum fails to induce Fas up-regulation in cultured endothelial cells, and direct contact between FasL⁺ CD8⁺ T cells and Fas-expressing vascular wall cells has not been observed in human KD specimens

Of note, despite the cytotoxic-related transcriptional signatures observed in peripheral blood CD8⁺ T cells and in single-cell studies, perforin and granzyme expression was not clearly detected in CD8⁺ T cells within human coronary artery lesions [53]. This discrepancy highlights the important gap between peripheral cytotoxic transcriptional programs and demonstrated effector function within coronary tissue. Future research should focus on validating these cytotoxic pathways in human samples and exploring alternative mechanisms of T-cell-mediated vascular damage to bridge this translational gap. The hypothesized cytotoxic pathways as well as the translational discrepancy between murine KD-like models and human pathological observations are summarized in Fig. 3.

### Noncytolytic Inflammatory Injury Triggered by CD8^+^ T Cell-Derived Cytokines

Beyond direct cytotoxicity, these CD8⁺ T cells​ exert noncytolytic effects by secreting IFN-γ and TNF-α, which continuously regulate the functional states of vascular endothelial cells [34, 61]. In vitro studies have shown that IFN-γ can reduce nitric oxide and cyclic guanosine monophosphate (cGMP) levels in endothelial cells and increase endothelial permeability, whereas TNF-α can impair endothelial barrier function by affecting the cytoskeleton and cellular oxidative state [83, 84]. Concurrently, TNF-α induces endothelial hyperpermeability through cytoskeletal reorganization, including actin stress fiber formation and disruption of tight junction proteins via RhoA/ROCK signaling pathways [85–87]. In the acute phase of KD, the serum levels of IFN-γ and TNF-α are significantly increased, and are closely related to the disease activity and the occurrence of macrophage activation syndrome [88]. Previous studies have shown that IFN-γ and TNF-α can affect endothelial cell permeability and the nitric oxide signaling pathway. However, in Kawasaki disease, dysregulation of these cytokines is thought to be involved in the pathological process of coronary artery lesions [64, 89].

**Fig. 4:**
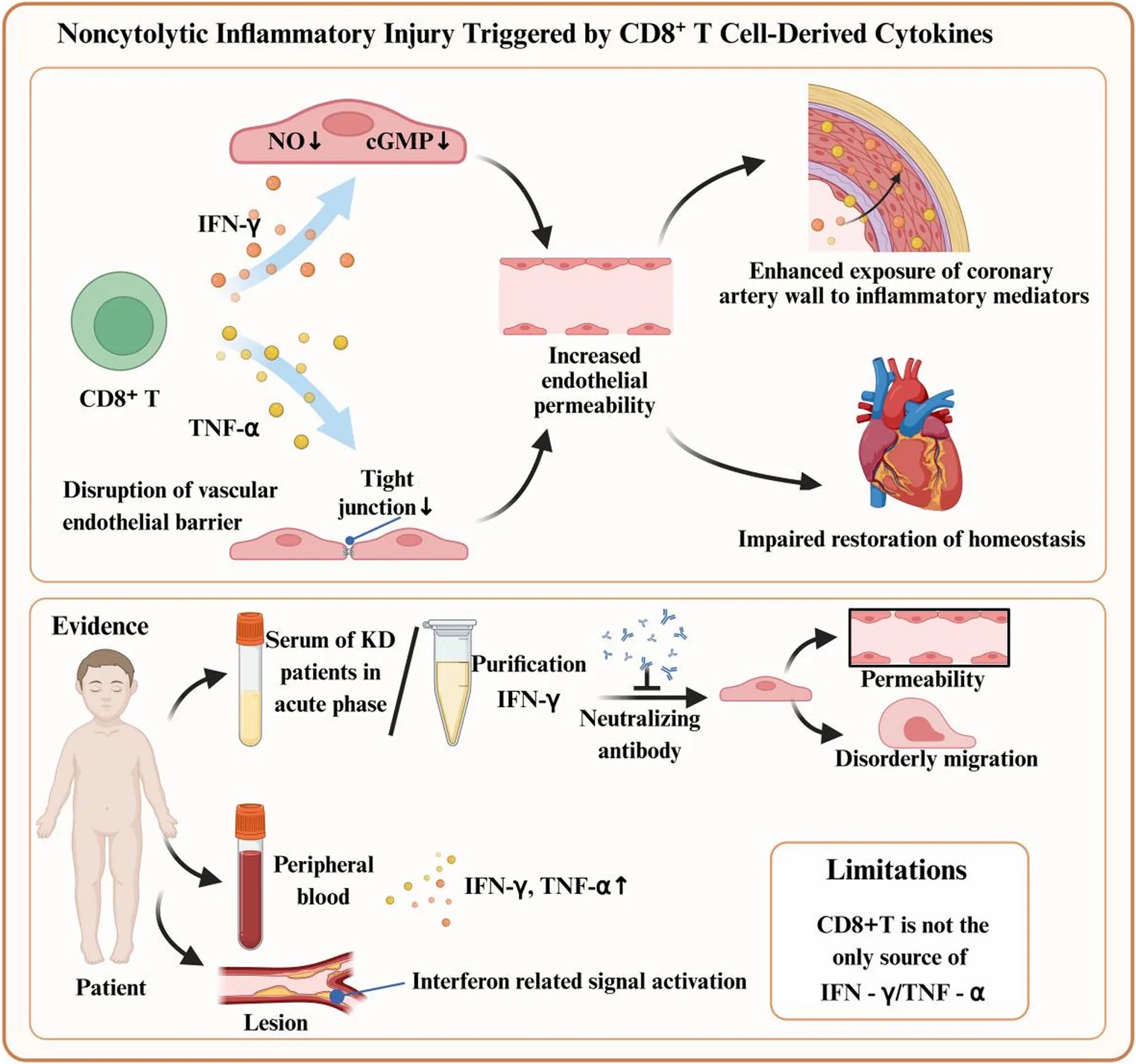
Non-cytolytic inflammatory injury triggered by CD8⁺ T-cell-derived cytokines in Kawasaki disease. Schematic depicting non-cytolytic vascular injury induced by CD8⁺ T-cell-derived IFN-γ and TNF-α. Secreted IFN-γ reduces endothelial NO and cGMP levels, while TNF-α disrupts tight junctions and reorganizes the endothelial cytoskeleton, jointly increasing endothelial permeability and impairing vascular barrier integrity. Damaged endothelium facilitates exposure of the vascular wall to circulating inflammatory mediators and hinders post-inflammatory homeostatic restoration. Clinical observations show elevated circulating IFN-γ and TNF-α in acute KD patients, which correlate with coronary artery lesion development. In vitro neutralizing-antibody experiments further confirm the contribution of IFN-γ to an abnormal endothelial migratory phenotype induced by acute-phase KD plasma. It should be emphasized that CD8⁺ T cells are not the exclusive cellular source of IFN-γ and TNF-α during KD-related systemic inflammation

Existing evidence shows that cytokines such as IFN-γ and TNF-α can affect the functional state of vascular endothelial cells and participate in the vascular inflammatory process of Kawasaki disease [90]. Clinical data further showed that the serum levels of IFN-γ and TNF-α in children with KD were significantly increased and positively correlated with the occurrence of coronary artery lesions, suggesting that endothelial damage mediated by this axis is the core link in the progression of the disease [91, 92]. Elevated circulating levels of IFN-γ and TNF-α during the acute phase correlate with the development of coronary artery lesions and resistance to intravenous immunoglobulin therapy in KD patients [93, 94]. Furthermore, plasma from children in the acute phase enhances HUVEC migration, an effect attenuated by anti-IFN antibodies, suggesting that IFN-related signaling drives dysregulated endothelial migratory behavior under inflammatory conditions [90]. These cytokine-mediated disruptions compromise vascular endothelial integrity, potentially increasing the exposure of the coronary artery wall to inflammatory mediators and interfering with the restoration of homeostasis after inflammation subsides [95]. Persistent endothelial barrier dysfunction following the resolution of acute inflammation may contribute to long-term vascular remodeling and late-onset coronary complications in KD survivors. While current evidence highlights the role of noncytolytic CD8^+^ T cell​ functions in pediatric vasculitis, further research is needed to delineate the specific molecular mechanisms linking cytokine-induced endothelial modulation to chronic cardiovascular sequelae. The non-cytolytic injury cascade driven by CD8⁺ T-cell-derived pro-inflammatory cytokines is depicted in Fig. 4.

### Sustained Vascular Inflammation and Coronary Structural Remodeling

Coronary artery lesions in KD may involve three interrelated pathological processes: acute necrotizing arteritis, subacute or chronic vasculitis, and luminal myofibroblastic proliferation [96]. Acute necrotizing arteritis typically manifests within the first 2 weeks after the onset of fever and is predominantly mediated by neutrophil infiltration, which can lead to saccular aneurysm formation, thrombosis, or vascular rupture [96]. This initial inflammatory response is so strong that it makes the wall of the blood vessel weak, and can lead to future long-term complications if not treated in a timely and appropriate manner. The subacute and chronic phases, on the other hand, are characterized by infiltration with lymphocytes, mainly CD8^+^ T cells, and can last for several months to years [97]. While the direct role of CD8⁺ T cells in initiating acute necrosis remains unclear, evidence suggests they are critical in maintaining persistent local inflammation during the later stages of vascular injury.

Considering the infiltration of CD8⁺ T cells into affected coronary artery walls and the persistence of T-cell-related inflammatory signals in chronic lesions, CD8⁺ T cells may be more involved in maintaining subacute/chronic vasculitis, whereas their direct role in acute necrotizing arteritis remains unclear [41, 80]. Constant vascular inflammation has been related to the dysregulated expression of the matrix metalloproteinases (MMPs) and tissue inhibitors of metalloproteinases (TIMPs) that degrade the extracellular matrix and elastic lamina [42, 98]. This proteolytic activity/inhibitory imbalance is also an important factor in the structural instability of the coronary artery wall leading to the aneurysmal dilatation and weakening [99, 100]. Meanwhile, pathological myofibroblasts that are formed through the proliferation of vascular smooth muscle cells lead to abnormal repair and progressive stenosis of the lumen and thrombosis [96]. This fibroproliferative response represents a maladaptive healing mechanism that transitions the vessel from an inflammatory state to one of obstructive remodeling.

Although timely treatment has reduced severe outcomes, long-term follow-up indicates that cardiovascular events and mortality remain concerns, albeit at low incidence rates, highlighting the need for continued surveillance [99]. Future research should focus on elucidating the specific molecular triggers linking CD8⁺ T cell persistence to MMP/TIMP dysregulation to develop targeted therapies that prevent both aneurysmal and stenotic sequelae.

## Discussion and Perspectives

### Current Evidence and its Limitations

Current studies have investigated the role of CD8⁺ T cells in KD mainly at three levels: human peripheral blood, coronary artery pathology, and animal models. Different types of evidence have distinct research objectives and scopes of interpretation [10, 15, 41, 45, 71]. Human peripheral blood studies can characterize dynamic changes in CD8⁺ T cell​ numbers, activation status, subset composition, transcriptional features, and TCR repertoires over the disease course and following treatment, and currently represent the most suitable type of study for longitudinal follow-up [23, 45, 50, 101]. Nevertheless, peripheral CD8⁺ T cell proportions and absolute counts are subject to numerous confounders: shifts in other immune populations, cellular redistribution, proliferation, apoptosis, and therapeutic interventions [45, 46, 50]. Accordingly, a reduction in circulating CD8⁺ T cells does not directly indicate impaired function, and cytotoxicity-, proliferation-, or exhaustion-related transcriptional features observed in peripheral blood do not necessarily reflect the actual functional state of these cells within affected coronary arteries [41, 45, 64, 65]. In addition, differences among existing human studies in study populations, disease stages, sampling times, cell-subset classification criteria, and detection methods limit direct comparisons of their findings.

Human coronary artery histopathological studies provide relatively direct spatial evidence that CD8⁺ T cells enter local lesions; however, tissue infiltration alone cannot establish that these cells initiate vascular injury or determine their specific modes of action [41, 79, 80]. Histopathological analyses of human coronary artery lesions consistently demonstrate infiltration by CD8⁺ T cells, particularly in fatal or severe cases, suggesting their central role in local inflammation [72, 102]. Previous pathological studies did not detect clear expression of perforin or GZMB in CD8⁺ T cells infiltrating coronary artery lesions, indicating that classical perforin–granzyme-mediated direct cytotoxicity has not yet been demonstrated in coronary artery injury in human KD [82]. However, these infiltrating CD8⁺ T cells typically lack detectable expression of perforin and granzyme B (GZMB), indicating that classical perforin-granzyme-mediated direct cytotoxicity may not be the primary driver of vascular injury in KD. Furthermore, the available coronary artery tissue samples are limited in number and are predominantly derived from fatal cases or patients with severe or chronic coronary artery lesions, and may therefore primarily reflect the immunopathological characteristics of severe disease or specific disease stages [41, 80, 96]. Beyond the coronary arteries, infiltration of CD8⁺ T cells and macrophages has also been observed in mitral valve tissue from one child with KD-associated severe mitral regurgitation, although the generalizability of this finding remains unclear [103].

The LCWE model has provided functional intervention evidence that CD8⁺ T cells promote the development of coronary arteritis through cell-depletion and adoptive-transfer experiments [71]. However, an extensive immune repertoire analysis of these animal models shows that there are major differences between these models and human KD, especially in terms of the patterns of clonal expansion, B-cell response, and antigen-specific adaptive immunity of patients [104]. Functional studies are based on LCWE-induced murine models and have shown evidence for CD8^+^ T cells in the development of coronary arteritis in the form of cell-depletion and adoptive-transfer experiments [105].

Moreover, the inducing agent itself may be relevant. Most KD-like mouse models, including the widely used LCWE model, rely on bacterial cell wall components, whereas a preceding clinically apparent infection is not documented in a substantial proportion of human KD cases. CD8^+^ T-cell activation in infection-associated and non-infection-associated KD may therefore involve different antigenic drivers, and this distinction should be considered when extrapolating findings from animal models to human disease [106]. Future human studies should attempt to stratify patients according to the presence or absence of a recognized infectious trigger when evaluating the role of CD8^+^ T cells [107].

### Clinical Application Value

The immune characteristics of CD8⁺ T cells may represent candidate biomarkers or associated immune phenotypes related to IVIG non-responsiveness, coronary artery lesion risk, and disease activity; however, no mature indicator is currently available for direct clinical decision-making, and predictive performance with independent validation has not been demonstrated. Previous studies have suggested that pretreatment HLA-DR-related CD8⁺ T cell​ activation phenotypes are associated with IVIG non-responsiveness, providing preliminary support for their inclusion in treatment-response risk assessment [23, 108]. In addition, a longitudinal study found that, in the IVIG-nonresponsive group, the absolute number of CD8⁺HLA-DR⁺ T cells was relatively low before treatment and increased significantly after the initial IVIG treatment, whereas no significant change was observed before and after treatment in the IVIG-responsive group [109]. However, the current findings mainly reflect statistical associations and fail to establish that these indicators can independently and consistently identify children with IVIG non-responsiveness. They may therefore be more appropriately applied as complementary indicators together with clinical characteristics and conventional inflammatory markers. Their predictive and monitoring value requires further validation in larger sample sets and independent cohorts.

CD69 and CD25 are frequently used markers of early T-cell activation. In acute KD, increased CD69 expression on peripheral CD8⁺ T cells indicates prominent early activation, and CD25/CD69 changes may reflect immunoregulatory processes, including activin A-mediated regulation [44]. However, their independent value for predicting IVIG resistance or coronary artery lesion formation has not been established; the relevant studies are limited by small sample sizes, in vitro designs, variable sampling times, and the lack of validated thresholds. These markers should therefore currently be regarded as candidate biomarkers requiring validation in larger, well-characterized cohorts.

In addition, longitudinal changes in other CD8⁺ T-cell activation markers may also be useful for assessing immune status after treatment. An early study found that the proportion of CD8⁺CD38⁺ T cells was lower during convalescence than before IVIG treatment, although the direction of early changes after IVIG treatment was inconsistent among individual patients [110]. Together with the above-mentioned HLA-DR-related findings, current evidence suggests that CD8⁺ T-cell activation states change dynamically with disease progression and treatment, and persistence or elevation of certain activation phenotypes may reflect incomplete resolution of post-treatment immune abnormalities. However, heterogeneity across studies in activation markers, outcome measures and sampling times makes it difficult to define a consistent dynamic pattern with clear clinical significance, and standardized testing time points and diagnostic thresholds are lacking. Overall, CD8⁺ T cell​ surface activation phenotypes may help evaluate IVIG response and post-treatment immune status, yet their clinical application requires further validation in multicenter prospective studies. Beyond surface activation phenotypes detectable by flow cytometry, the transcriptional and clonal features of CD8⁺ T cells may also provide new candidate biomarkers for identifying immune states associated with coronary artery lesions [45, 64, 65, 68]. Should these findings be validated in independent cohorts and translated from complex molecular features into simplified and reproducible testing panels, their applications may be further extended to immune phenotyping, risk identification for coronary artery lesions, and stratification of treatment responses.

From a translational therapeutic perspective, the functional evidence provided by the LCWE model suggests that CD8⁺ T-cell-related immune processes may have potential value for therapeutic research [71]. However, CD8⁺ T cells play essential roles in anti-infective immunity and immunological memory formation; accordingly, non-selective depletion of the entire CD8⁺ T cells compartment is not suitable for direct clinical intervention. Future studies should identify CD8⁺ T cell subsets driving coronary inflammation and their key effector pathways, and validate associations between these features, vascular injury and treatment outcomes using human peripheral-blood and coronary-lesion tissue samples. On this basis, selective modulation of aberrant CD8⁺ T cell​ responses may deliver greater translational therapeutic value than broad suppression of total CD8⁺ T cell​ populations.

### Future Research Directions

Future work should establish multicenter prospective longitudinal cohorts with standardized inclusion criteria, sampling time‑points, cell-subset gating criteria and detection assays. Such standardized frameworks can validate the associations between dynamic changes in CD8⁺ T-cell counts, activation status, functional subsets and both IVIG response and coronary outcomes. In addition, it is imperative to build stronger translational links between peripheral-blood observations and coronary-tissue evidence. Given limited access to human vascular lesions, paired blood-tissue analyses are needed to identify pathogenic CD8⁺ T-cell subsets involved in vascular inflammation and characterize their crosstalk with myeloid and vascular wall cells. These combined efforts will improve mechanistic insights into KD-related coronary injury and facilitate the development of risk-assessment tools and targeted therapeutic strategies.

## Conclusion

In this review, we summarize the current understanding of CD8^+^ T-cell involvement in coronary injury in KD by converging evidence from the single-cell omics, TCR repertoire analysis, human pathology and animal models, and peripheral blood profiling. Acute KD is accompanied by dynamic disturbances in peripheral CD8^+^ T cell​ homeostasis including a numerical loss, increased activation, altered subset distribution and the development of proliferative, cytotoxic or exhaustion-related transcriptional profiles and TCR clonally expanded CD8^+^ T cells. Pathological studies confirm infiltration of the coronary lesion by CD8^+^ T cells and animal models suggest that they can induce coronary arteritis. Such lymphocytes can infiltrate inflamed vessels and maintain vascular inflammation and remodeling through both​ cytotoxic and non-cytotoxic mechanisms. However, with limited access to human vascular specimens, the subset of pathogenic cells and the in-vivo effector functions in human KD are not fully understood.

CD8^+^ T-cell-driven immune responses are an important mechanistic association between systemic inflammation and coronary damage in KD. These gaps in knowledge are likely to be addressed by future investigations with prospective cohort data, paired blood-tissue analysis and functional assays, which could lead to the creation of new biomarkers for coronary risk stratification and immunotherapies to target the disease without compromising the protective immunity of the host.

## Data Availability

No datasets were generated or analysed during the current study.
